# Gene-environmental influence of space and microgravity on red blood cells with sickle cell disease

**DOI:** 10.1038/s41525-024-00427-7

**Published:** 2024-09-30

**Authors:** Norris E. Igbineweka, Jack J. W. A. van Loon

**Affiliations:** 1 Imperial College London, Centre for Haematology, Department of Immunology & Inflammation, Commonwealth Building, Hammersmith Campus, Du Cane, London, W12 0NN UK; 2 Department of Haematology, King’s College Hospital NHS Foundation Trust Denmark Hill, SE5 9RS London, UK; 3 Dutch Experiment Support Center (DESC), Department of Oral and Maxillofacial Surgery/Oral Pathology, Amsterdam Bone Center (ABC), Amsterdam UMC Location VU University Medical Center (VUmc) & Academic Centre for Dentistry Amsterdam (ACTA), Gustav Mahlerlaan 3004, 1081 LA Amsterdam, The Netherlands; 4 European Space Agency (ESA), European Space Research and Technology Centre (ESTEC), TEC-MMG, Keplerlaan 1, 2201 AZ Noordwijk, The Netherlands

**Keywords:** Genetics, Haematological diseases

## Abstract

A fundamental question in human biology and for hematological disease is how do complex gene-environment interactions lead to individual disease outcome? This is no less the case for sickle cell disease (SCD), a monogenic disorder of Mendelian inheritance, both clinical course, severity, and treatment response, is variable amongst affected individuals. New insight and discovery often lie between the intersection of seemingly disparate disciplines. Recently, opportunities for space medicine have flourished and have offered a new paradigm for study. Two recent Nature papers have shown that hemolysis and oxidative stress play key mechanistic roles in erythrocyte pathogenesis during spaceflight. This paper reviews existing genetic and environmental modifiers of the sickle cell disease phenotype. It reviews evidence for erythrocyte pathology in microgravity environments and demonstrates why this may be relevant for the unique gene-environment interaction of the SCD phenotype. It also introduces the hematology and scientific community to methodological tools for evaluation in space and microgravity research. The increasing understanding of space biology may yield insight into gene-environment influences and new treatment paradigms in SCD and other hematological disease phenotypes.

## Introduction

Gene-environment interaction is a complex field of study which is presently expanding. How a single gene mutation interacts with variable environmental stimuli to modulate expression of a phenotype is pertinent to that of both hematological disease and human biology^1,2^. Can environmental variations be measured and quantified in a meaningful way to predict clinical heterogeneity and disease severity? One disease model that could yield biological insight under novel environmental stressors like that of space and microgravity is sickle cell disease (SCD) due to its clinical variability between affected individuals despite being a monogenic disorder. Can space medicine provide further understanding of clinical complications within the context of SCD? Would an environment of space and microgravity allow for further delineation of genetic signatures and bio-physiological parameters that can predict early who will get severe disease or end-organ damage before they become clinically manifest? In this upcoming era of multiple novel treatments for SCD (both pharmacological and curative), can this novel environmental stimulus pave further understanding of personalized medicine and who likely will respond to which treatments? The advent of new treatments like Voxelotor in SCD affecting the allosteric characteristics of hemoglobin causing oxygen dissociation curve to shift to left such that oxygen affinity is increased and therefore retained, will be interesting to explore under microgravity. Consequently, space medicine could offer a host of distinctive possibilities to further dissect and characterize genotype-phenotype relationship as well as elucidate novel cellular kinetics in SCD.

## Sickle cell disease pathophysiology

SCD is a monogenic disorder involving pathological hemoglobin polymerization leading to ‘sickling’ of erythrocytes^3^. Key bio-rheological properties of sickling include less red cell deformability and increasing stiffness where intracellular sickle cell hemoglobin (HbS) is proportionally related to degree of cell stiffness^3–7^. Hemolysis, another key pathological feature of the disease, is associated with the irreversible structural change to sickle red blood cells^5,8^. Other major bio-mechanical properties of sickled erythrocytes are increased blood viscosity^9^ and cell adhesiveness^10^. These properties culminate in blockage of the microvasculature termed vaso-occlusion leading tissue ischemia, organ damage and ongoing sickle vasculopathic damage^3,4,11^. In addition, recently, the classical concepts of ’ineffective erythropoiesis’ have had novel importance in SCD under hypoxic conditions and new markers of ineffective erythropoiesis recently developed^12,13^. While there are many known environmental influences to SCD pathophysiology, additional environmental stressors of microgravity on SCD pathophysiology have yet to be explored.

## Biological modifiers in sickle cell disease

### Biomarkers of HbS polymerization

In addition to the quantitative amount of intracellular HbS, two other major biomarkers that influence degree of HbS polymerization and SCD clinical severity are fetal hemoglobin (HbF), and co-inheritance of a-thalassemia^14^.

RBC with increased HbF have longer survival compared to cells with increased HbS; and elevated HbF is well known to inhibit HbS polymerization^15,16^. HbF is a key regulator of SCD clinical course with elevated HbF associated with more ameliorated SCD outcomes^17^. Different SCD haplotypes have varying levels of HbF with Senegal and Arab-Indian β-globin gene-like cluster haplotypes tending to have higher HbF and therefore milder SCD clinical symptoms^18^. Bantu haplotypes have the least HbF levels and therefore worst clinical outcomes which include a three-fold risk of cerebrovascular accident (CVA), kidney injury, chronic lung disease with cor pulmonale, leg ulcers, as well as young adult death^18^. Genetic modifiers that regulate HbF have also been discovered over last two decades and show significant association with clinical course in SCD^19^. Three Quantitative Trait Loci (QTL) contributes to 20–50% of HbF common variation— BCL11A on chromosome 2p, HBS1L-MYB intergenic polymorphism (HMIP) on chromosome 6q11, and Xmn1-HBG2 (rs7482144) on the β-globin cluster on chromosome 11p^20–22^. Recently, a fourth HbF-associated gene FRMPD4 has been found in an X-linked locus that could explain a significant fraction of Fcells% variation in SCD^23^.

The co-inheritance of α-thalassemia regulates SCD as the defected production of alpha globin chains, reduces polymerization through lowering of total intracellular HbS^14^. Co-inheritance of α-thalassemia is observed in 1 in 3 individuals with SCD and offers an attenuated effect to the natural SCD course with prolonged survival, protection against complications like CVA and cholelithiasis^24,25^.

**Fig. 1:**
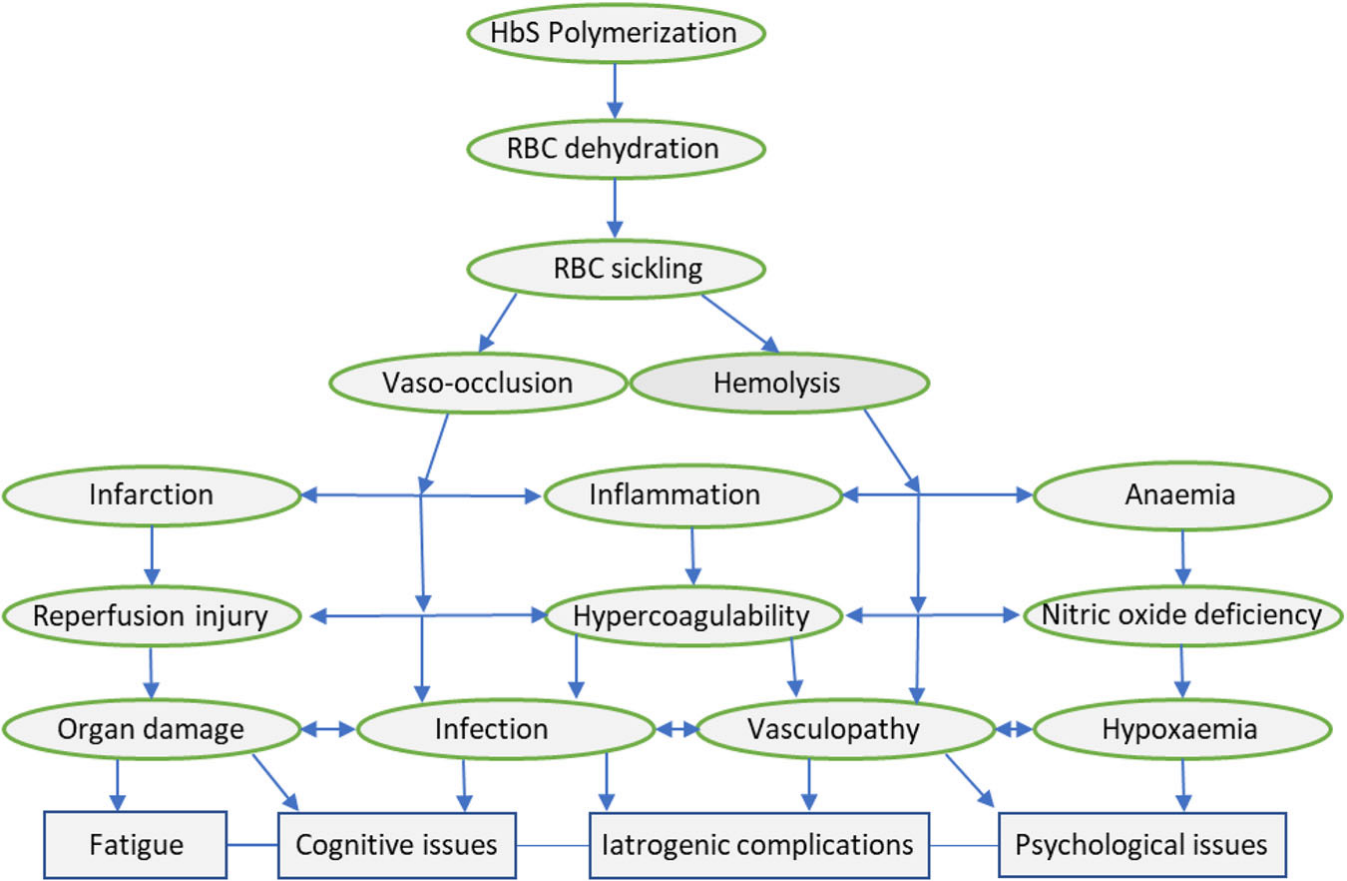
Summary view of SCD pathophysiology emanating from HbS polymerization (adapted from Rees et al.^195^). An overview of the complex and inter-related pathophysiological processes in SCD emanating from HbS polymerization.

Other biomarkers that influence HbS polymerization is RBC dehydration caused by increased cation permeability leading to less RBC deformability^26^. Increased pain crises were found in patients with SCD that correlated with increased RBC dehydration and dense cells (defined as >1.11 mg/mL of hemoglobin) thereby increasing sickling propensity^27^. RBC membrane disruption in SCD leads to exposure of erythrocyte phosphatidylserine and phosphatidylethanolamine^14^. Consequently, there is increased RBC adhesion to the vascular endothelium through binding of reticulocyte receptor CD36 (Glycoprotein IV) to thrombospondin on the endothelial lining of blood vessels and binding of reticulocyte receptor integrin α4β1 (very late activation antigen-4) to both vascular cell adhesion molecule-1 (VCAM-1) and fibronectin on blood vessel endothelial lining^28^. This process culminates in vaso-occlusion and pain crises ultimately cascading to organ failure (see Fig. 1)^14,28^. Studies have shown that expression of CD36 and integrin α4β1 is conversely reduced in SCD reticulocytes when Hydroxycarbamide (Hydroxyurea; a disease-modifying SCD therapy), is used^28^. SCD endothelial adherence is also a marker of disease severity^29^.

### Biomarkers of hemolysis

Hemolysis is a cardinal clinical manifestation in SCD which contributes to pathogenesis and variability in clinical severity^25^. Vascular dysfunction, and systemic vasculopathy, with subsequent pulmonary hypertension, chronic kidney disease, large-artery ischemic stroke, leg ulcers, and priapism are characteristic of the hemolysis phenotype. Cell-free hemoglobin inactivates nitric oxide (NO) through a dioxygenation reaction which oxidizes hemoglobin to methemoglobin (a non-oxygen-binding hemoglobin form which readily loses heme). Circulating free hemoglobin and heme constitute erythrocytic danger-associated molecular pattern (eDAMP) molecules, activating the transition of the endothelium and innate immune system to a pro-inflammatory and pro-adhesive state^25^. This clinical state leads to SCD vaso-occlusion and acute lung injury in SCD murine models^25^. Impaired NO bioavailability from intravascular hemolysis leads to altering redox balance, and oxidative stress. This negatively influences physiological alterations which govern inflammation, hemostasis, blood flow and angiogenesis^25^.

Low hemoglobin, as a result of hemolysis, is a robust biomarker of hemolysis in SCD and is associated with unfavorable outcomes including abnormally high tricuspid regurgitant jet (TRJ) velocity^30^, abnormal transcranial Doppler (TCD) velocities^31^, stroke secondary to cerebral infarction or cerebral hemorrhage^32^, and premature death^32,33^. Increased serum lactate dehydrogenase (LDH) is also associated with high TRJ and abnormal TCD velocities as well as priapism and leg ulcers in patients with SCD^14^. LDH is also increased with apoptosis and premature RBC death in erythropoiesis^25^.

### Biomarkers of inflammation and oxidative stress

The inflammatory component in SCD is also a key contributor to HbS polymerization. Elevated expression of leukocyte adhesion molecules such as L-selectin and αMβ2 are associated with SCD pain crises states^34^. High total white blood count (WBC) is indicative in severe clinical SCD states such as acute chest syndrome (ACS), brain infarcts and death^35^. Total WBC reduction is also one of the mechanisms of therapeutic effect with Hydroxycarbamide use^35^. Other inflammatory components such as interleukins (IL2, IL3, IL6, IL8, and IL10), prostaglandin-E2, and tumor necrosis factor-α are elevated in SCD^36^. C-reactive protein (CRP) is elevated in vaso-occlusion leading to acute painful crises or ACS^37^. The secretory phospholipase A2 (sPLA2) enzyme produces fatty acids by cleaving phospholipids which trigger a cycle of inflammatory events^38^. Increased levels of sPLA2 can predict onset of ACS with 100% sensitivity and specificity of 67%^38^. A small sample, randomized clinical trial showed that blood transfusion, another SCD therapeutic strategy, reduces levels of sPLA2 and thereby reduces ACS events^39^.

In addition to cell-free hemoglobin, free heme released from free hemoglobin following oxidation, is also a powerful source of oxidative stress^25^. Nonetheless, free heme does not directly scavenge NO. Free heme mediates inflammation and vessel injury. For example, in mice with SCD, free heme promotes inflammation, coagulation, and vaso-occlusion, effects of which can be inhibited by the heme scavenger hemopexin^40^. In cell culture, heme augments high levels of secretion of placental growth factor which subsequently causes the release of the powerful vasoconstrictor endothelin 1, which is a known mediator of pulmonary hypertension^41,42^.

There are various other physiological oxidants which include hemoglobin oxidation, which react with NO^43–45^. In SCD, there is heme-related propagation of eDAMPs which act through the triggering of innate sterile inflammatory components such as TLR4 and NALP inflammasome signaling. These pathways lead to acute rises in endogenous (HMGB1) and exogenous (LPS) ligands of TLR4 which in turn activate production of reactive oxygen species, and neutrophil extracellular traps (NETs). These mechanisms also augment oxidative stress and further impair the redox balance^46^. These pathological systems are likely magnified in a vaso-occlusive crises^25^. There is small evidence to suggest that adenosine, an endothelial vasodilatory mediator like NO, can bind to RBC receptors, leading to increased 2,3-diphosphoglycerate (DPG) and sphingosine-1-phosphate, which is linked with lower oxygen affinity of hemoglobin^25^.

Recently, there has also been growing body of evidence demonstrated in SCD through implication of the gut microbiome with significant gut microbiome dysbiosis observed in bacteria that are known to have strong pro-inflammatory triggers^47^. There is also some evidence that this gut microbiome dysbiosis can lead to persistent pain through changes in vagal nerve activity^48^.

### Biomarkers of hypercoagulability

Biomarkers of hypercoagulability in SCD are complex and multifactorial. Even in steady, non-crises SCD state, there is aberrant activation of the fibrinolytic system, elevated levels of thrombin generation markers, increased expression of tissue factor and reduced levels of natural anticoagulants^49^. The coagulation process is activated with the enhanced exposure to erythrocyte phosphatidylethanolamine, membrane erythrocyte phosphatidylserine and circulating microparticles from erythrocytes, white blood cells, endothelial cells, and platelets therefore SCD is thought of as a heritable thrombophilia^50^. Free heme induces tissue factor expression on the endothelial surface lining in blood vessels.

### Biological markers of rheological RBC properties

Abnormal rheological properties such as increased red cell rigidity has aggravating consequences to HbS polymerization in SCD^51^. This is because cell deformability is the capacity of a body to alter its shape in response to a deforming force. This is a primary determinant of blood dynamics. It is important that a fully functional RBC should pass effectively through the microcirculation with no impedance. The most irreversibly sickled RBC are the cells with the least cell deformability^51^.

Factors which contribute to RBC deformability are categorized into intrinsic i.e., related to internal contents: (i) viscosity, (ii) elasticity, (iii) fragility, (iv) increased cation permeability, (v) cell water loss, and (vi) stiffened cytoskeleton^51^. Extrinsic factors relates to that of volume, surface area and environment which in turn includes factors such as: (i) osmolality, (ii) oxygen tension, (iii) pH, (iv) temperature, and (v) plasma protein concentration^51,52^. Worsening SCD mechanical parameters has been demonstrated to correspond with dehydration and increased viscosity secondary to increased intracellular hemoglobin concentration^51,52^. Cell sickling secondary to deoxygenation augment membrane permeability to cations (K+, Na+, and Ca2+) chaperoned by water efflux leading to dehydration^53,54^. Hydration status determines surface area-volume relationship. If there is overhydration of a sickle cell, then its volume increases. Nonetheless, surface area remains unchanged as biologically a 3% to 4% increase in surface area culminates in cell lysis with overall reduction in cell deformability^51,55^. Diametrically, dehydration increases cellular concentration of hemoglobin and therefore cellular viscosity, culminating to reduced deformability also^56,57^.

Abnormal SCD membrane properties secondary to abnormal hemoglobin polymer formation causes a displacement of membrane phospholipids and proteins. This culminates in the characteristic SCD morphology^58^. SCD membranes differ bio-mechanically from normal RBC membranes in both their static and dynamic rigidities^57^. The unique usual morphological properties of the RBC allow for amplified flexibility and optimized surface area in order to navigate microvascular networks^54,55^. These healthy RBC have malleable cytoskeleton, excess membrane, and homogeneous intracellular fluid of hemoglobin protein. This promotes the high deformability needed for functional RBCs to squeeze through microvasculature and participate in oxygen/carbon dioxide exchange amongst tissues^51^. In SCD, the polymerized sickle hemoglobin have reduced cellular deformability and increased viscosity. In addition, repeated cycles of sickling and unsickling, reduce the RBC lifespan to less than 20 days in the blood circulation, in comparison to the 120-day lifespan of a healthy RBC^59^. Consequently, in SCD, there is a chronic anemia, which in turn, leads to bone expansion in volume and hematopoietic activity reflecting stress erythropoiesis and abundance of immature erythrocytes (reticulocytosis)^60^.

Fluid mechanics and macro-rheology also influences SCD pathophysiology. Blood viscosity is representative of intravascular flow resistance. This can be viewed as ratio of the force of resistance against flow to the velocity of flow (viscosity = shear stress/shear rate)^51^. Shear stress is a tangential drag force per unit area as the viscous blood flows across a vessel wall. Viscosity is a pre-determining factor of shear stress^51^. The SCD RBC has a predisposition to form aggregates which is shear dependent. This also has variable impact on viscosity. High shear conditions tend to disrupt aggregates. In comparison, low shear conditions tend to promote aggregate conditions and thereby increasing viscosity^61^. Increased blood viscosity is probably the most consequential patho-biomechanical aberrancy in SCD together with dense cells and are both key determinants of SCD severity^51^.

Some of these markers can be measured. For example, SCD deformability can presently be evaluated by ecktacytometry and microfluidic techniques^62^. In ecktacytometry, a small whole blood sample is mixed with the highly viscous polyvinylpyrrolidone (PVP) solution. This is then transferred into the space between a rotating cup. With cup rotation, the RBC in suspension undergo varying shear stresses depending on the cup rotational speed. Deformable RBCs then elongate in reaction to the high shear rates. This is due to the difference in viscosity of PVP solution compared to the Hb solution inside of the cells^63^. Lasered light directed at the suspension produces a diffraction pattern from which the elongation index (EI) is calculated (EI = (length – width)/(length + width))^62^. The EI is a measure of RBC deformability in ektacytometry. This provides an average assessment of the rheological properties of the RBC population as a whole as opposed to individual measurements. An increased EI indicates better RBC deformability. This is associated with ameliorated clinical parameters in SCD and correlated with improved EI & RBC deformability parameters in patients with SCD on Hydroxycarbamide and blood transfusion^64,65^. Microfluidic platforms are devices that can be used to model the effects of deoxygenation on RBC rheology and viscosity, factors of which impede blood flow in SCD^66^.

Blood viscosity is determined by: (i) plasma viscosity, (ii) hematocrit, (iii) RBC deformability, (iv) RBC aggregation, and (v) altered oxygen status. These factors contribute to %dense RBC as measured by ADVIA hematology analyzer (Siemens, Tarrytown, NY, USA)^67^. These factors also lead to SCD vaso-occlusion^68,69^. The importance of blood viscosity is highlighted by the fact that individuals with SCD with a hematocrit of 21% would have a comparable whole blood viscosity with someone without SCD with a hematocrit of 45% even in normoxia^9^. Blood viscosity can also be defined as hematocrit to viscosity ratio (HVR). This correlates with RBC oxygen capacity and adjusts for differences in hematocrit between individuals^70^. Higher HVR is associated with better oxygen carrying capacity^70^. Lower HVR is associated with SCD complications such as leg ulcers^68,71^. Given correlation with SCD phenotypes and disease severity, it will be beneficial to evaluate and measure SCD rheological properties under various altered environmental/disease states as well as within therapeutic monitoring. SCD rheology therefore altogether reflects important functional parameters in SCD pathogenesis. The Lorrca® ektacytometer with Oxygenscan (RR Mechatronics, Zwaag, The Netherlands) can measure RBC deformability under various oxygen concentrations & deoxygenated states and represents a new phase of advancing technology. The EI when RBCs are oxygenated, have less HbS polymers present. This point is associated with highest RBC flexibility (EImax)^72^. The EI is when RBCs are deoxygenated, and is peak HbS polymerization. This point corresponds to least RBC deformability (EImin)^72^. The point of sickling is the oxygen tension (pO2) to which the RBC begin to sickle^72^. The EImin, EImax, and point of sickling (PoS) vary between individuals and can be modulated therapeutically^73–75^.

### Ineffective erythropoiesis

Recently, the classical concepts of ’ineffective erythropoiesis’ have had increasing novel importance in SCD and markers of ineffective erythropoiesis have recently been developed^12,13^.

El-Hoss and colleagues^12^ were able to model the hypoxic environment of the bone marrow and found that death of sickle erythroblasts secondary to hypoxia began at the polychromatic stage, and these were positively selected cells with high HbF levels. Cell death was linked with cytoplasmic sequestration of heat shock protein 70 which was rescued by HbF induction synthesis. The study was able to provide evidence for ineffective erythropoiesis in SCD and demonstrates an anti-apoptotic role for HbF during terminal erythroid differentiation. Brewin et al.^13^ evaluated a novel quantitative index for measuring ineffective erythropoiesis (IoIE) in homozygous (HbSS) SCD. Potential mechanisms for ineffective erythropoiesis include increased HbS autooxidation, free iron that induces reactive oxygen species production, structural membrane defects with increased phosphatidylserine (PS) exposure, and lower affinity of alpha chain coupling with HbS leading to unbalanced alpha and beta chain coupling^76^.

## Treatments targeting biomarkers of sickle cell disease

Two US FDA approved pharmacological agents for SCD, Hydroxycarbamide; and L-glutamine, both affect hemorheological parameters in SCD^62,77,78^. As well as the known induction of HbF which ameliorates SCD-related complications, HU also causes beneficial reduction in WBC, adhesive reticulocytes to endothelium possible via reduction of Lu/BCAM phosphorylation and reduction in inflammation^79,80^. Patients with SCD on HU typically gain an increase in hemoglobin by approximately 1 g/dL which does increase the whole blood viscosity; but this is offset by the greater rheological improvements to the SCD RBC^81^. HU-therapy has been associated with reduction in % dense RBC six months after HU treatment had been initiated and HU-therapy has also been associated with reduced number of irreversibly sickled cells^82,83^.

L-Glutamine was shown, in a phase III clinical trial, to reduce painful VOC, number of hospitalizations, and duration of hospital stay in SCD^84^. Nevertheless, there were no changes in the clinical laboratory parameters^85^. Clinical amelioration is thought to be attributed to lessened oxidative stress due to increase in the reduced form of nicotinamide adenine dinucleotides in RBCs of patients with SCD^62^. It is believed that L-glutamine may also be of benefit through improving rheological properties the SCD RBC^62^. SCD RBC adhesiveness is also reduced with use of L-glutamine; albeit these studies were performed in static adhesion assays which do not always replicate SCD physiological in vivo state^62^.

Other existing SCD treatment modalities are allogenic hematopoietic stem cell transplantation (allo-HSCT) and blood transfusion. Allo-HSCT can provide a curative strategy for SCD management and has an overall survival of >90%^86^. Nevertheless, HSCT has limited matched donor availability of only 10-15% of the SCD patient population^87^. Other complications surrounding HSCT include graft vs. host disease (GVHD), graft rejections, organ toxicities and potential sterility^86^. Additionally, although HSCT is a curative measure, if a patient with SCD has existing irreversible organ damage at baseline, irregular blood flow can persist, even after undergoing HSCT^87^. Blood transfusion can have the direct beneficial hemorheological effect of decreasing HbS % and simultaneously improving RBC deformability^88^. Blood transfusions also increases hemoglobin and therefore lead to improvement in oxygen carrying capacity. On the other hand, this also means that blood transfusion can increase hematocrit and blood viscosity, and if not done carefully, can potentially have negative consequence in SCD^88^.

Regarding newer therapies, Voxelotor (GBT440-007; Global Blood Therapeutics, San Francisco, CA, USA) is a novel agent in the SCD therapeutic landscape which inhibits HbS polymerization by stabilizing the R-state hemoglobin conformational state as well as increasing hemoglobin-oxygen affinity^89^. In the phase I/II clinical trial, adolescents who were receiving Voxelotor improved hemoglobin by 1 g/dL as well as reduced hemolytic markers such as bilirubin and reticulocytes^90^. Patients with SCD receiving Voxelotor also showed persistent reduction in irreversibly sickled RBCs beyond 90 days of treatment^91^. Given hemoglobin rise following Voxelotor use, it would be anticipatory to expect deleterious effects from increased viscosity. Nevertheless, Voxelotor is associated with improved SCD RBC deformability and decrease in viscosity as shown in experimental studies in vitro^92^. Directly lowering viscosity with Voxelotor have yet to be shown in vivo^62^. However, it seems that similar to HbF, when Voxelotor is present and bound to at least 30% of hemoglobin molecules, the greater HbS becomes less deoxygenated and polymerized which is the more predominant effect compared to an increase in viscosity^93^.

Another new therapeutic agent in SCD is Crizanlizumab (SEG101; Novartis, Basal, Switzerland), a p-selectin monoclonal antibody, which works by inhibiting p-selectin interaction with leukocytes^94^. The 12-month SUSTAIN clinical trial demonstrated efficacy and safety in patients with SCD both on and off HU. Crizanlizumab therapy was shown to lower rates of VOC compared to placebo as well as increased the duration of time to first crises^94^. There were no significant changes in hemoglobin, reticulocytes, LDH and indirect bilirubin were observed between Crizanlizumab and placebo groups suggesting that the benefits of Crizanlizumab are not directly related to inhibition of hemolysis or intrinsic RBC changes^94^.

One of the other exciting therapies for SCD demonstrated in hemoglobinopathy clinical trials are pyruvate kinase activators such as Etavopivat which demonstrate pleiotropic mechanistic effects toward reducing RBC sickling including through reducing 2,3-diphosphoglycerate (2,3-DPG) which thereby increases hemoglobin (Hb)-oxygen affinity. The augmented binding of oxygen reduces HbS polymerization and sickling^95,96^. PKR (pyruvate kinase in RBC) activation elevates levels of adenosine triphosphate (ATP) generated through a glycolytic flux, thus re-energizing the RBC, and leading to preservation of membrane integrity, improvement in RBC deformability, reducing hemolysis and demonstrating exciting potential as a novel disease-modifying agent for SCD^97^. Pyruvate kinase gene, PKLR, has historically shown significant genetic diversity in malaria endemic populations, in particular, African populations, with some of these variants associated with reduced falciparum malaria. Recently, novel intronic PKLR variants have been associated with acute pain in SCD^98^.

Gene therapy is an additional novel potentially curative strategy for patients with SCD. In some respects, it extends access to stem cell transplant techniques who are particularly limited by matching donors as donor cells, in this case, come from the patient themselves^99^. In this process, the patient undergoes harvesting for hematopoietic stem and progenitor cells, these cells are then modified using lentiviruses or genome editing, and then transplanted back into the patient. The two main approaches include gene therapy (lentivirus based) and gene editing (CRISPR-Cas9 based)^100^. Lentiviral-based approaches have been utilized for β-hemoglobinopathies which work either by increasing HbF induction through introduction of lentiviral constructs containing a γ- or by a hybrid β-promotor/γ-globin gene^99^. The second way is by introducing a mutated β-globin that interferes with HbS to prevent HbS polymerization^99^. For example, the BB305 LentiGlobin (Bluebird Bio, Cambridge, MA) is a self-inactivating lentivirus which encodes an “anti-sickling” β-globin. Published reports of this approach in a 13-year-old with SCD showed a total hemoglobin of 11.7 g/dL, 2% HbF and ~47% HbAT87Q^101^.

Other gene editing strategies are clustered regulatory interspaced short palindromic repeats and associated Cas9 (CRISPR/Cas9) system which allow for favorable editing rates at 85–90% after 16 weeks in human bone marrow cells^102^. CRISPR Therapeutics and Vertex Pharmaceuticals have adopted this approach in their clinical trials.

## Environmental modifiers of sickle cell disease

SCD pathogenesis is, however, also highly governed by environmental stimuli such as hypoxia, pH, and temperature which can precipitate hemoglobin polymerization and vaso-occlusion^3,4,11,103^. Endogenous cellular environmental transitions include that of normoxia to hypoxia, isotonic to hypertonic, acidotic to alkalotic, macro- to microcirculation, and laminar to turbulent flow milieus are critical factors to variability of the sickle erythrocyte influencing its pathophysiology^25^.

External environmental factors, include meteorological-specific, such as wind speed, air pollution, ozone, carbon monoxide, nitric oxide, altitude, weather and particulate matter have all been demonstrated to have a strong influence on factors of SCD phenotype such as incidence of hospitalization with painful vaso-occlusive crises (VOC)^103–110^. Although, certain environmental and genetic modifiers of SCD are known, no complete bio-predictor profile of disease severity is available yet and clinical course remains highly variable from patient to patient. There remains no approved pharmacological cure by the European Medicines Agency (EMA) nor by the United States Food and Drug Administration (FDA), with treatment response to present-day disease-modifying agents variable amongst patients with SCD.

SCD has had a longstanding intricate relationship with its environment. Before the advent of modern biology, SCD was known to local African communities who observed individuals with increased sickle pain in rainy seasons and termed it ‘rainy season rheumatism’ in their local dialects^3,4^. Cooler weather has long been described as precipitant to painful VOC in SCD. However, any extremes of temperature i.e., very hot temperatures can also precipitate crises^103^. Geographic areas where the sickle mutation is prevalent, also coincide with environments of malarial endemicity.

The ontology of sickle cell gene mutation origins itself suggest that selective pressure of malarial endemicity could have pre-dated the sickle gene mutation development of a sickle mutation^111^. Due to historical and ongoing population dynamics, SCD is a pressing global health issue; nonetheless 80% of SCD burden resides in Africa^112^. High mortality still affects Africa where 50–90% of children with SCD die before reaching their 5th birthday^113,114^. This is juxtaposed to the West in countries with advanced health systems, patients with SCD are living longer but still lag 30 years behind the wider general population^115^.

There have been case studies of exertion-related deaths amongst individuals with sickle cell trait when doing military basic exercises, mountaineering, high-intensity sports-related activities as well as the environmental impact of heat and humidity^116^. The pathophysiological mechanisms of sudden unexpected death amongst individuals with sickle cell trait is put down to four categories: (i) heat stroke & exertional heat illness; (ii) exertion-related cardiac arrythmia; (iii) exertional related rhabdomyolysis; and (iv) exertion-related acute syndrome^103,116,117^. These factors would be certainly useful to understand in novel environmental stressors like that of microgravity and spaceflight. As new frontiers in studying the influence of space and microgravity on sickle erythrocytes open up, it is more likely that healthy astronauts or passengers with sickle cell trait will precede SCD patients into space. This serves a unique opportunity for individuals with sickle cell trait to pave the way of understanding to whether those possessing the SCD genotype can enter spaceflight, if necessary, protecting actual patients with SCD from entering spaceflight, if deleterious to do so. It is also a chance to stress test RBC with sickle cell trait in itself, to delineate the effect of the environmental influence of space and microgravity as akin to exertion-related pathology associated with sickle cell trait.

A study of 1047 hospital admissions of acute pain in adults and children with SCD, also showed that high wind speed (26–57 knots) was significantly associated with more hospital admission compared to low wind speed (4–16 knots)^108^. These findings have been replicated in three other countries: USA, Canada, and France^104–107^. Studies have shown that high levels of ozone have been associated with increased hospital admissions for acute pain^109,110^. Although some studies have identified the opposite effect with high levels of ozone being associated with lower rates of acute pain admissions^107,110^. There have also been suggestions that ozone may be able to increase the rate and capacity of oxygen absorption in erythrocytes which could potentially ameliorate complications in SCD^107,110^. Nitric oxide is a known potent vasodilator and lack of nitric oxide has long been implicated in SCD pathogenesis, such as vasculopathy and SCD-related complications like pulmonary hypertension, leg ulcers, and priapism^103,108^. In the retrospective London study of 1047 admissions, higher levels of nitric oxide were associated with less acute painful crises^103,108^. Parallel to nitric oxide exposure, carbon monoxide is also beneficial to SCD through its vasodilator, anti-inflammatory, and cytoprotective properties. Carbon monoxide also shifted the hemoglobin oxygen dissociation curve to the left. In addition, carbon monoxide prolongs the half-life of SCD erythrocyte and reduces microvascular stasis through reduced NF-κB which leads to anti-inflammatory properties in transgenic SCD mice^103,108^.

Exposure to particulate matter (fine: PM2.5, coarse: PM10) has also been implicated in causing health issues in non-sickle populations as well as in SCD populations^103^. It is estimated to be responsible for approximately 3,000,000 deaths per year and is most endemic in cities, where in conjunction, the incidence of SCD is highest^103^. In SCD, these fine particles can enter the bloodstream and damage the endothelium which is already central to SCD vasculopathic process. In SCD natural history, there is increased risk of cerebrovascular disease^103^. However, particulate matter is associated with added stroke risk. Furthermore, exposure to both PM2.5 and PM10 was associated with increased daily admission amongst patients with SCD in Parisian hospitals^107^. These findings were also seen with PM10 in a Sao Paulo hospital in Brazil^109^.

High altitude has also been associated with increased frequency of SCD complications particularly in mountainous areas and in aircrafts^103^. For example, the rate of acute VOC is double in individuals living in the highlands compared to those that lived in the lowlands. Those in the highland also had 5% increase in hemoglobin to which increased blood viscosity leads to worsened clinical outcomes in SCD^11^. A US study identified a 6.5% risk of developing acute VOC on commercial flights. This initially pre-empted advice to individuals with SCD to travel with supplementary oxygen during each flight^103^. However, it is now observed that individuals with SCD rarely experience complications with commercial flights and oxygen supplementation or pre-flight transfusion should only be used if there are pre-existing complications such as cerebrovascular or pulmonary-related. SCD, due to its pathogenesis and intricate relationship with its environment, can provide an intriguing model to explore effects related to exposure of microgravity. This may in turn provide novel insight toward ameliorating its disease severity as well as improving understanding of biology of humans in space.

## Hematological effects of spaceflight/microgravity and methodological tools for conducting space medicine genomics research to assess impact of microgravity on the hematopoetic system

### Spaceflight impact on hematological parameters, erythroid transcription factors, and chromatin structure

Recently published in a Nature Scientific Report^118^, Japanese investigators, while characterizing the impact of spaceflight on secondary lymphoid organs, found out that the erythrocyte-related gene transcription factors GATA-1 and Tal1 from splenic cells were significantly downregulated in mice exposed to orbital space and simulated gravity of 1 g with an in-flight centrifuge compared to ground control mice. Spleen size and mass was also reduced in the mice exposed to both in-flight microgravity and centrifuge^118^. A recent transcriptomic study of astronauts to and from long-distance spaceflight aboard the International Space Station (ISS), revealed leukocyte transcriptome changes in response to the adaptive immune modulation associated with spaceflight^119^. At a cellular level, gravity has therefore been shown to influence differentiation, proliferation, function, gene expression, signaling and chromatin structure^120^. In addition, chromatin structure carries importance for SCD also, where forced chromatin looping from adult β-globin to the fetal γ-globin promoter can lead to augmented reactivation of γ globin expression^121^. This further increases fetal hemoglobin levels, a key biological modulator that ameliorates clinical severity in SCD^121^.

### Hemolysis and anemia associated with spaceflight

Anemia has persistently been a pathological feature associated with space missions and had been characterized by a reduction of 10–12% of volume^122^. Nevertheless, underlying mechanisms behind all of this had been prior unclear and ascribed to changes to plasma volume, hemoconcentration and low erythropoietin (EPO) levels within the first 10 days upon entering space which did not provide full mechanistic understanding^123^.

Recently, published in Nature Medicine paper, Trudel^124^ and colleagues tried to unpick some of these mechanisms associated with spaceflight anemia. The authors found that by utilizing an improved accurate and sensitive space-permitting endogenous alveolar CO monitoring on 14 astronauts (11 men and 3 women), ongoing hemolysis (independent of EPO) was detected both with head-down tilt bed rest microgravity simulation^125^ and in actual spaceflight which served as explanation for the observed anemia^124^. CO monitoring which provided for serum analytes (haptoglobin, bilirubin, iron, transferrin percent saturation, ferritin, C-reactive protein and EPO); as well as a blood sample for full blood count and reticulocytes. It was further deduced that hemolysis was a primary effect of microgravity in spaceflight and not an acute adjustment to hemodynamic alterations upon entering microgravity^124^. The authors therefore advocated for spaceflight to be seen as a ‘hemolytic condition’ that should be recommended for screening and monitoring of both astronauts and space tourists^124^.

So, a crucial question here is, what would be the outcome should other hemolytic conditions such as SCD and beta-thalassemia undergo exposure to spaceflight and conditions of microgravity or (chronic) hypergravity?

The mechanism and anatomic site for the space hemolysis remains undetermined^124^. It is hypothesized that ineffective erythropoiesis could play a factor in the marrow adipose tissue accumulation as there is a paucity of bone marrow stimulation in space microgravity conditions^126^.

There is altered shape and size of erythrocytes and spleen in space^118,127^. It is also thought that the lifespan of erythrocytes may be shortened by mitochondrial dysregulation and stress that is associated with spaceflight^128^. Genetic modifiers of HbF such as BCL11A regulate expression of globin genes and this regulation involves alterations of methylation patterns on certain DNA regions to contribute to expression^129,130^. Ineffective erythropoiesis in thalassemia is linked with DNA hypomethylation in the β-globin gene cluster^130^. Analogous to this, is that modified methylation levels were found on CD4 and CD8 lymphocytes one year after spaceflight although this date should be taken with care since this is only a case study^123^. Consequently, it might be postulated whether space, microgravity or radiation could have effects on DNA methylation of regulatory regions modulating globin expression and gene modifiers that could contribute to underpinning space hemolysis. In addition, future studies should also consider the effects of space on targeted drugs that improve hemolysis and anemia such as HbF inducers like Hydroxycarbamide, PK activators, as well as the microgravity effect on allosteric qualities on drugs like Voxeletor.

Contributing mechanisms for anemia are that microgravity does also reduce commitment to erythrocytic lineages^131^. When hematopoietic clusters of differentiation-34+ (CD34+) cells were exposed to simulated microgravity for 2 days and actual microgravity for 11 days, they demonstrated decreased proliferation and differentiation^132^. Myeloid progenitor cell counts increased while erythroid progenitor cell counts decreased in microgravity with erythroid progenitor cells showing disrupted cell cycle progression and migration potential of these cells under microgravity^133^.

### Spaceflight and oxidative stress

Recent data also show that simulated microgravity imposes stress, both oxidative and osmotic, on cellular function^134^. In SCD pathogenesis, oxidative stress is an important contributing factor to worsening phenotype^135^. It will be pertinent to investigate the extent of oxidative injury with planned experiments in microgravity both simulated and true. The study of SCD in microgravity will allow for further characterization of erythroid morphology and kinetics, thus providing baseline knowledge for future long duration space missions. It will also further give insight into the bio-regenerative hematopoeitic capacity while in space. The ultimate benefit of space-related research is the possibility for improved understanding of cellular mechanisms and regenerative cell pathways which can translate toward biochemical responses applicable to diagnostic medicine and rehabilitation.

Space-related biological research will, on a human level, yield crucial insight into gravity-related and adaptation responses within the human body while in space such as around stress response, DNA repair, and genetic aberration. Finally, it will also provide necessary data needed toward whether humans that possess copies of the sickle cell gene mutation (both carrier state and disease form) can enter spaceflight. Through companies like Virgin Atlantic and Blue Origin and developments in spaceflight over recent years, we have entered a new dawn where spaceflight is becoming more commercialized for the routine use of the general public. As in the early days of the aviation industry, commercial tickets are presently priced high at $250,000 per healthy astronaut/passenger at Virgin Galactic and $35,000 per healthy passenger for NASA^136^. Eventually, however, spaceflight will become affordable to most citizens. As such, it will become pertinent to understand the physiological burden of space microgravity for a particular disease state for medical and insurances purposes. Precipitants of sickle cell polymerization like hypoxia and dehydration are highly sensitive to environmental factors such as high altitude. As such, data for individuals with SCD in space will be much needed. In doing so, we will obtain a more intricate clinical understanding of SCD in addressing questions like what SCD phenotype will predominate in space i.e., vaso-occlusive or hemolytic? Will there be a more frequent number of vaso-occlusive crises? What is the type of SCD chronicity and long-term complications which can be modulated by spaceflight? Can the interaction between space environment and SCD genotype/phenotype reveal any translational insights that will be useful for prognostic, disease-modifying and therapeutic mechanisms?

### Genotoxic damage

Recently, the role of DNA damage to the hematopoietic system and predisposition to leukemia through mechanisms of endogenous and environmental genotoxic aldehyde damage have been further elucidated^137^. This has not fully been explored in SCD and if studied, could reveal interesting insights. This is relevant firstly in the context that two patients with SCD were diagnosed with leukemia subsequent to gene-therapy^138^ and secondly, some patients with SCD are being treated with HU, a known cytotoxic agent, but is not directly mutagenic or directly binding DNA; but rather it’s genotoxic effect is restricted to indirect clastogenicity in certain cell types only when time and high dose thresholds are surpassed^139^. Finally, these observations would be useful to evaluate in SCD in environment of real microgravity, since a growing body of evidence show there is DNA damage particularly from cosmic radiation in space which can cause DNA damage in blood cells^140^.

It has been clearly shown that biological systems such as animal and cell models display altered function and behavior in different gravity conditions^1,141^. Similarly, in simulated microgravity, it is clear that cells react to weightlessness in an altered manner also and response seem dependent on the cell type in question^1,142^. While the response of different cell types have been described, the intracellular regulatory pathways involved in genetic expression, epigenetic mechanisms, stem cell differentiation and cross-talk remain poorly understood. This makes research in conditions of space and microgravity extremely promising^1,2^.

### Methodological tools for conducting space medicine genomics research and assessing impact of microgravity on the hematopoietic system

Here are some of the methods used to simulate microgravity environments which can be applied for the study of the hematopoietic system and SCD research.

#### In vitro models

##### Random positioning machines

Random positioning machines (RPM) are devices which allow for simulated microgravity on exposed hematopoietic cells^143,144^. RPMs have been demonstrated to be useful and show good correlation between simulated and real microgravity environments for human leukemic myelomonocytic cell lines^145^, monocyte^146^, and T-lymphocyte populations^147^. For example, Villa et al.^145^ using simulated microgravity with RPMs, the authors demonstrated reduced proliferation of the human leukemic myelomonocytic cell line U937. The same findings of the reduced growth of the U937 human leukemic myelomonocytic cell lines, were also observed in a prior space shuttle experiment^148^. Similarly, locomotion capacity of monocytes has been shown to be impaired in both simulated microgravity RPM studies as well as actual space studies possibly secondary to altered cytoskeletal structures^146^. RPMs themselves have freely rotating constructs which are driven by two motor-frames and simulate a microgravity environment with gravity vector averaging^149^. The main RPM device limitations include the generation of a fluid shear^150–152^ or inertial shear^143^.

##### Rotating wall vessels

Rotating wall vessels (RWV), also referred to as rotary cell culture systems (RCCS), are liquid-filled containers which slowly rotate and are NASA approved simulator for microgravity^149,153^. RWVs rotate continually which leads to a vector averaged microgravity^143^. The rotation speed at which this occurs is related the specimen’s viscosity, specific gravity, and vessel fluid density^131^. Alternative designs to the RWVs are these slow turning lateral vessels (STLV)^154^ and high aspect ratio vessels (HARV)^155^ which are essentially just variations in shape and size^156^. RWV has been utilized for the evaluation of B-lymphocytes in relation to Epstein-Barr virus (EBV) infection were it was observed that there was significantly lower expression of proteins associated with lytic cycle and suppression of EBV reactivation in lymphoblastoid cells cultured in simulated microgravity^157^. This also corroborated trends in actual space environments where for humans, B-lymphocytes occupy the peripheral circulation in humans, commonly in its own separate gravitational state such that gene expression may be distinct from that typically seen in a monolayer cell culture^157^.

##### Clinostats

Clinostats experiments have also played a role in providing new hematological insight. For example, Wang et al.^158^ demonstrated that clinorotation led to upregulation of inducible nitric oxide synthase (iNOS) in an AP-1-dependent manner and this was a pathway for postflight orthostatic intolerance. In SCD, there is aberrant nitric oxide depletion, through the release of cell-free hemoglobin worsening hemolysis, vasoconstriction and overall vasculopathy making this pathway an intriguing line of study^159^. Clinostats have a tube-like composition which is filled with a cell suspension and then rotated to simulate exposure to microgravity^160^ Some important clinostat parameters include rotation speed and tube volume size which govern the proportion of centrifugal force necessary to stave off driving cells toward the vessel walls^149^. The main limitations to clinostats are issues of tube width as well as smaller sample volume and size^131^.

##### Large diameter centrifuge

Large Diameter Centrifuges (LDC) allows for the examination of parameters of hypergravity (any value above Earth 1 g—where g is the gravitational acceleration at the surface of the Earth)^161^. In a centrifuge, gravity is created by an inertia force such that an object in motion will move at the same speed in the same direction unless imposed to change its direction^162^. A centrifuge represents this action by forcing the object to repeatedly change directions thus generating a centripetal, acceleratory force like the force on mass on Earth causing unit gravity. In order to separate the effects of weightlessness from other variables associated with spaceflight and manipulate gravity as an experimental variable, a centrifuge can provide a means to study various gradients of gravity and hypergravity experimentally. Centrifuges can also be applied to explore microgravity via the Reduced Gravity Paradigm^163^. LDC has been shown to be an effective model of study in plasma and endothelial cells^164,165^. In the case of endothelial cells, brief periods of microgravity and hypergravity both led to distinct result patterns. LDC simulated microgravity led to reduced endothelial cell barrier integrity whereas simulated hypergravity led to improvement in the endothelial cell wall integrity which was mediated through NO^165^.

The LDC at European Space Agency/European Space Research and Technology Centre (ESA/ESTEC) (Noordwijk, The Netherlands) has utilized this for experiments in life and physical sciences over recent years. The diameter of the LDC is eight meters. It has four arms, each of which can support two gondolas with a maximum payload of 80 kg per gondola. In practice, up to six gondolas are used for an experiment plus one additional gondola in the center for control/reference purposes. The LDC can execute experiments that last from one minute up to six months, without stopping.

##### Levitation magnet

A levitation magnet can simulate near zero gravity^162^. Sub-atomically, in the physical world, all objects possess a magnetic force which could be counterbalanced to the force of gravity. The principles of the levitation magnet are that also biological objects, which are normally diamagnetic, can be levitated when placed in a strong magnetic field gradient^166^. Orientating this magnetic force in opposition to gravitational force gives a net force of zero on the object. Thus, magnetic levitation expels all stresses and pressures from gravitational forces and attempts to closely mimics orbital freefall^167^. However, the main disadvantage with this technology is the application of a large magnetic field that can also orient, especially biological system polymers in a certain non-physiological direction^168^. Tissue-types such as erythrocytes, also comes with slight variability that requires adjustment. Erythrocytes are iron-containing elements which may have some additional effect on the magnet. Nevertheless, levitation magnet is the only method to directly compensate for gravity on a ground-base at molecular level and has been used to as an effective model for simulated ‘weightlessness’ to study bone marrow cells as well as differentiated adipose and osteogenic cells^167,169^.

#### In vivo/animal models

##### Hindlimb suspension, partial weight bearing, skeletal muscle paralysis, and head-down tilt bed rest

Hindlimb suspension (HLS), partial weight bearing (PWB), skeletal muscle paralysis (SMP), and head-down tilt bed rest are all frequently used in vivo models for microgravity study. HLS is a model which exposes a rodent’s hindlimbs to unloading through tail suspension^170^. This leads to musculoskeletal unloading of the hindlimbs triggering a cephalic fluid shift similar to that encountered by astronauts during spaceflight. Nevertheless, a key limitation of HLS is that often these rodents demonstrate signs of increased stress associated with weight loss compared to rodents in ground control^170^. This therefore makes differentiation of effects challenging and interpretation of results from HLS-microgravity experiments problematic. HLS has been used to simulate a space and microgravity environment to investigate the hematopoietic system in mice where the authors found a reduction in mature RBCs (bone marrow; peripheral blood) and B-lymphocytes (bone marrow; spleen) as well as increased populations of macrophages and monocytes^171^. The cytoskeleton of bone marrow‐derived mesenchymal stromal cells (BM‐MSCs) was perturbed in addition to the upregulation of the expression hematopoietic-related genes such as granulocyte-macrophage colony-stimulating factor, tyrosine kinase-3 ligand and interleukin-3^171^.

Animal models such as mice with SCD e.g., Berkeley/Townes mice will be a critical component of ongoing studies in understanding the influence of space and microgravity on SCD RBC as such methodological approaches will likely take place long before actual individuals with the SCD genotype make it to space.

PWB is an alternative model of suspension where the total body of the animal is suspended and therefore the weight borne by each limb is reduced^172^. PWB is actually a beneficial model as it can more closely simulate conditions of hypogravity akin to the Moon at 0.16 g or Mars at 0.38 g^172^. This model does, nonetheless, significantly reduces muscle mass and bone density^173^. PWB has been used to investigate simulated space and microgravity models on blood counts where it has been found statistically significant differences in total white blood cells (WBC), neutrophils, monocytes, lymphocyte, and eosinophils within the first two days of experimental exposure to PWB in mice^174^. These changes in leukocytes have also been consistent with findings observed in spaceflight^175,176^.

SMP also induces musculoskeletal unloading with swift musculoskeletal loss of tissue in one of the hindlimbs through the use of neurectomy (spinal cord injury) or Botox^177^. The neurectomy allows for atrophy to occur in both bone tissue and skeletal muscle, as well as disturbance of the immune and cardiovascular systems akin to astronauts on long periods of spaceflight^177^. A newer upgrade of this is single limb immobilization through casting which also leads to a lot of bone loss and skeletal muscle loss as observed during exposure to microgravity environments^178^. Nonetheless, this model is still being perfected and very small amount of studies having been undertaken to evaluate its efficacy. Both SMP and HLS studies have largely been focused on the musculoskeletal systems. In addition, the cephalic fluid shift and bone loading from ground reaction forces seen with HLS, does not occur for limb immobilization techniques of paralysis like neurectomy, botox and casting^131^.

The 6-degree head-down tilt bed rest model also attempts to mimic microgravity through the induction of musculoskeletal disuse and reproduces the cephalic fluid shifts experienced during spaceflight upon tilted incline^179^ but also dry emersion is used as analog for human spaceflight^180^. Through microgravity simulated 6-degree head-down tilt bed rest studies, anemia via the mechanism of low-grade hemolysis was importantly delineated^125^.

There are also possibilities to expose humans to hypergravity using a Short Arm Human Centrifuge (SAHC)^181^. SAHC are used mainly to develop countermeasures to reduce microgravity-related pathologies^182^. SAHC mainly have been used in the field of cardiovascular sciences, for example, to study the effects of rotational axis position on peripheral blood displacement (central hypovolaemia) and cerebral perfusion^183^. These type of centrifuges have even been used to treat patients with cardiovascular disorders like lymphoedema, Raynaud’s phenomenon, Peripheral and Coronary artery disease (CAD/PAD) and Complex regional pain syndrome (CRPS)^184^.

#### Parallel in vitro and in vivo models

##### Parabolic flight

Parabolic flights give short-term freefall by temporarily curtailing the ground reaction forces which provides microgravity exposure^185^. Aircraft parabolic flights are useful in performing experiments of limited duration under conditions of reduced gravity as actual spaceflight itself can be infrequent, costly and have limited availability of capsule space, as well as extraordinary cost^162^. Their main value is to allow for quality and validation-related tests to improve the success of experiments in actual spaceflight. Sometimes parabolic flights may be sufficient and answer the research question as a stand-alone. Normally the aircraft are used to provide microgravity but there are also flights where Mars and Moon g’s are flown^153^. Parabolic airplanes can be divided into three groups: (a) large airplanes permitting dozens of passengers and a number of large experiments; (b) medium-sized airplanes which are used for single experiment with several operators and/or subjects; and (c) small airplanes and for single passengers and small experiments^162^. Parabolic flights are very useful for demonstrating qualitative ‘look-and-see’ phenomena, experiments for quantitatively measure of effects of gravity and also measuring transient phenomena from high g to low g^162^. The microgravity conditions are simulated with parabolic flight maneuvers to which there is steady horizontal flight where the aircraft ascends at approximately 45^0^ (the pull-up) for 20 s with accelerations between 1.8 g and 2 g. All aircraft thrust is then firmly reduced for approximately 20 to 25 s and the parabola the plane dives at about 45^0^ (the pull-out), accelerating at about 1.8 to 2 g for another 20 s followed by a steady horizontal flight again^162,186^. A recent parabolic flight study consisting of 15 parabolas with a Mudry CAP-10B acrobatic aircraft identified red blood cell aggregation as well as neutrophil and monocyte changes (particularly functional) in 6 healthy volunteers^187^.

Other, examples of parabolic flights include Novespace Airbus 310 ZERO-G which has a total flight duration of approximately 2.5 to 3 hours. Parabolas are flown in dedicated air zones over the Mediterranean Sea or the Gulf of Biscay^162^. Support for equipment design and all related safety aspects are provided by the French company Novespace based in Bordeaux and European Space Agency (ESA). The parabolic experiment takes place over two weeks with three flight days, which consist of 30 parabolas per day^162^.

##### Spaceflight

Known hematological effects during Spaceflight, for example, with reduction in hemoglobin mass, reduction in RBC mass and reduction in T-lymphocyte activity, have been well described since the mid-1970s^188,189^. Today, it is evidenced that just over 50% of spaceflight anemia is attributed to the mechanism of increased hemolysis as an exposure effect of space, particularly on astronauts as they continued through their lengthy missions^124^. Increased hemolysis by 54% was a primary effect of exposure to space in astronauts that persisted throughout their long-duration missions and may constitute the leading mechanism of space-related anemia^124^.

**Fig. 2:**
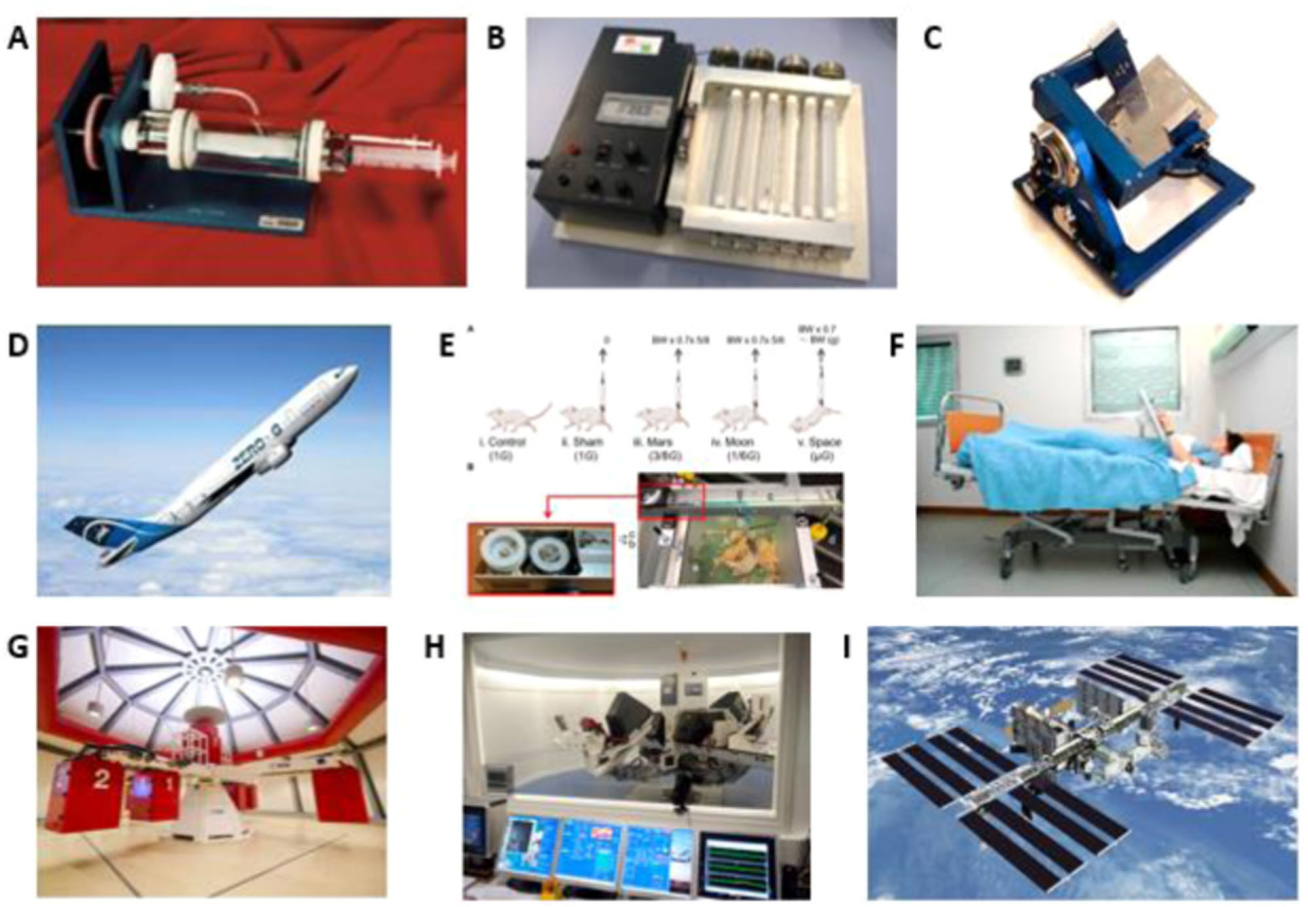
Summary of models for microgravity experiments. An overview for key models of microgravity experiments (images from National Aeronautics and Space Administration (NASA) and Synthecon Incorporated^TM^^196^, European Space Agency and European Space Research and Technology Centre (ESA-ESTEC)^197^, European Space Agency and Institut de Médecine et de Physiologie Spatiales (ESA-MEDES)^198^, Juhl et al.^131^, Zhang et al.^199^, and Novespace (France)^200^). **A** Rotating cell culture system (RCCS)^TM^ are slowly rotating liquid-filled containers^196^. **B** A fast rotating clinostat (ESA-ESTEC & Mary Ann Liebert incorporated)^197^, Noordwijk, Netherlands are tube-like composition filled with cell suspension and rotated to simulate exposure to microgravity. **C** Yuri Random positioning machines (Yuri RPM) Random positioning machines (RPM), freely rotating constructs driven by two motor-frames using gravity vector averaging to simulate microgravity. (ESA-ESTEC)^201^. **D** Parabolic flight (picture courtesy Novespace/France)^200^ give short-term freefall by temporarily minimizing ground reaction forces thus providing microgravity exposure as well as validating certain experimental tests prior to actual spaceflight. **E** Hindlimb suspension/partial weight bearing^199^ is an in vivo microgravity stimulatory study which unloads a rodent’s hindlimb through tail suspension. **F** 6° Head-down tilt bed rest (image: courtesy of MEDES/France)^202^, is an in vivo mimic of microgravity through the induction of musculoskeletal disuse and reproducing cephalic fluid shifts experienced during spaceflight upon tilted incline. **G** The Large Diameter Centrifuge at ESA-ESTEC (ESA/ESTEC)^203^. **H** The ESA Short Arm Human Centrifuge (SAHC) as located at the MEDES facility in Toulouse (France) (ESA/MEDES)^204^. **I** The International Space Station (ISS/NASA)^205^. ^TM^ (trademark).

In space, the situation of pure weightlessness is never encountered. Rather a mean low gravity (microgravity) situation is what is encountered which is approximately between 10^−6^ to 10^−4 ^g^162^. Consequently, although the devices and models discussed above generate an environment akin to conditions of microgravity they are all simulations and deviate with some aspects to real freefall conditions in orbital spaceflight^131^. Space launches for human missions can be with various systems from Kennedy Space Center in Florida, USA or from the Baikonur Cosmodrome. Preparation prior to space launch includes development of a Space Experiment Plan together with engineers, managers, and a team of scientific researchers. The experiment plan includes operational procedures such as monitoring, measurement, transportation, and storage of bio-samples. There is also compatibility validation testing and training of flight crew. Prior to launch, there are ground control experiments. Once spaceflight is complete, there is typically post flight review and analysis^162^. See Fig. 2 for a summary of microgravity experiments.

One of the newest platforms for microgravity research is the sub-orbital spaceflight (New Shepard and Virgin Galactic) particularly for human-related experiments^190^. This is essentially one big parabola incorporating several minutes of microgravity and permits research studies in between parabolic flights and orbital spaceflight experiments^190^.

## Discussion

Space medicine research is expected to provide expansive opportunities toward the understanding of SCD given its inherent susceptibility to environmental perturbations. This new approach may likely yield novel insights, through identification of novel aberrant molecular signals for example, that can facilitate improvement of knowledge toward SCD pathophysiology, therapeutics and answer the soon to be imminent question of whether individuals with SCD can enter spaceflight. These spaceflight and microgravity studies also offer doors of opportunity for study of other hematological conditions. In addition, spaceflight, could represent a pathophysiological process, in itself, to the haemopoietic system, which would require further elucidation.

SCD has long been the poster child for molecular medicine. From the 1940s, when chemist Linus Pauling defined the molecular basis of sickle cell anemia as the first molecular disease^191^, to Kan and Dozy in the 1970s defining the presence of single-nucleotide polymorphisms for the first time in the human genome in their paper entitled: ‘Polymorphism of DNA Sequence Adjacent to the Human Beta-Globin Structural Gene: Relationship to Sickle Mutation’^192^, to now the recent advance of gene-therapy strategies for SCD in present day^193^. SCD has also had wider impact globally and been on the forefront of great scientific insights toward our understanding of population genetics^194^. We should therefore seize this next chapter of SCD understanding for the advancement of the field through embracing our new frontier of space and microgravity. So up, up, and away to the stars, the time is now.
